# Bilateral Motor Cortex tDCS Effects on Post-Stroke Pain and Spasticity: A Three Cases Study

**DOI:** 10.3389/fphar.2021.624582

**Published:** 2021-04-21

**Authors:** Andrés Molero-Chamizo, Ángeles Salas Sánchez, Belén Álvarez Batista, Carlos Cordero García, Rafael Andújar Barroso, G. Nathzidy Rivera-Urbina, Michael A. Nitsche, José R. Alameda Bailén

**Affiliations:** ^1^ Department of Psychology, University of Huelva, Huelva, Spain; ^2^ Rehabilitation Area, Hospital Juan Ramón Jiménez, Huelva, Spain; ^3^ Autonomous University of Baja California, Mexicali, Mexico; ^4^ Leibniz Research Centre for Working Environment and Human Factors, Dortmund, Germany; ^5^ Department of Neurology, University Medical Hospital Bergmannsheil, Bochum, Germany

**Keywords:** anodal stimulation, fugl-meyer, post-stroke pain, primary motor cortex, spasticity, transcranial direct current stimulation

## Abstract

Stroke patients frequently suffer from chronic limb pain, but well-suited treatment approaches have been not established so far. Transcranial direct current stimulation (tDCS) is a safe and non-invasive brain stimulation technique that alters cortical excitability, and it has been shown that motor cortex tDCS can reduce pain. Some data also suggest that spasticity may be improved by tDCS in post-stroke patients. Moreover, multiple sessions of tDCS have shown to induce neuroplastic changes with lasting beneficial effects in different neurological conditions. The aim of this pilot study was to explore the effect of multiple anodal tDCS (atDCS) sessions on upper limb pain and spasticity of stroke patients, using a within-subject, crossover, sham-controlled design. Brain damage was of similar extent in the three patients evaluated, although located in different hemispheres. The results showed a significant effect of 5 consecutive sessions of atDCS, compared to sham stimulation, on pain evaluated by the Adaptive Visual Analog Scales -AVAS-, and spasticity evaluated by the Fugl-Meyer scale. In two of the patients, pain was completely relieved and markedly reduced, respectively, only after verum tDCS. The pain improvement effect of atDCS in the third patient was considerably lower compared to the other two patients. Spasticity was significantly improved in one of the patients. The treatment was well-tolerated, and no serious adverse effects were reported. These findings suggest that multiple sessions of atDCS are a safe intervention for improving upper limb pain and spasticity in stroke patients, although the inter-individual variability is a limitation of the results. Further studies including longer follow-up periods, more representative patient samples and individualized stimulation protocols are required to demonstrate the efficacy and safety of tDCS for improving limb symptoms in these patients.

## Introduction

Chronic limb pain and spasticity are common muscle symptoms in stroke survivors (Harrison and Field, 2015). Rehabilitation strategies usually aim to restitute motor functions by physiotherapy and physical rehabilitation. The effectiveness of these therapies and pharmacological treatment for reducing pain and spasticity is however limited at present, and alternative and complementary interventions are investigated to increase motor functionality and quality of life of these patients. Some of these new approaches are based on facilitation of neuroplastic changes that improve the physiological and functional recovery of post-stroke patients.

Several non-invasive brain stimulation (NIBS) techniques are available with potential to induce long-term potentiation like-plasticity associated with clinical and therapeutic effects, such as transcranial magnetic stimulation (TMS) (Simonetta-Moreau, 2014; Blesneag et al., 2015; D’Agata et al., 2016; Müller-Leinß et al., 2017) and transcranial electric stimulation (tES) (Boggio et al., 2007; Di Lazzaro et al., 2014; Kim et al., 2014; Darkow et al., 2017). In particular, transcranial direct current stimulation (tDCS) is a well-stablished tES method to induce neuronal excitability changes, including plasticity, by application of weak current (usually between 1 and 2 mA) through an anodal-cathodal electrode circuit through the scalp (Nitsche and Paulus, 2000; Nitsche and Paulus, 2001; Nitsche et al., 2005; Paulus et al., 2008; Bikson et al., 2010; Nitsche and Paulus, 2011; Antal et al., 2017). Considering that functional recovery of post-stroke motor symptoms is partially determined by neuroplasticity, modulation of cortical excitability of these patients by tDCS has emerged as an effective and well-tolerated therapeutic tool.

Beyond its effects on post-stroke motor function rehabilitation (Goodwill et al., 2016; Fregni et al., 2020), some exploratory clinical trials have shown also a beneficial effect of tDCS to reduce pain in several medical conditions, including stroke (Antal et al., 2010). Post-stroke pain has the characteristics of central pain. It depends on alterations of the spinothalamic tract and thalamocortical connections that affect primary motor (M1) and somatosensory cortex processing (Frese et al., 2006). Excitability-enhancing anodal tDCS over M1 can modulate activation of these pathways and the excitability of thalamic nuclei involved in pain processing (Fregni et al., 2006; Velasco et al., 2009). Therefore, anodal tDCS over M1 may be effective to reduce central post-stroke pain (Bae et al., 2014; Morishita and Inoue, 2016). Likewise, this approach enhances activity of the pyramidal system, and thus counter-balance lesion-based enhancements of muscle tone controlled by the extrapyramidal system (Simon and Yelnik, 2010). Therefore, by this mechanism, anodal tDCS might be suited to reduce post-stroke spasticity (Del Felice et al., 2016; Elsner et al., 2016; Leo et al., 2017; Levin et al., 2018). According to the cumulative effects of repeated sessions of M1 tDCS (Boggio et al., 2007; Sohn et al., 2013), facilitation of long-term potentiation like-cortical plasticity in stroke patients through multiple tDCS sessions should be a more effective method to treat motor symptoms, including pain and spasticity, than procedures based on one single session (Tanaka et al., 2011; Luedtke et al., 2012; Kindred et al., 2019), although lasting effects of one session approaches on pain have also been described (Bolognini et al., 2013; Kikkert et al., 2019).

Considering this background, we did explore the effect of anodal tDCS (atDCS) on upper limb pain and spasticity via a multi-session protocol (5 consecutive days), in which a bilateral electrode configuration (anode over M1 of the affected hemisphere and cathode over the contralateral M1) was evaluated in chronic stroke patients using a within-subject, crossover, double-blind, sham-controlled design. Thus, we aimed to identify the effects of anodal vs. sham tDCS on pain and spasticity in stroke patients not undergoing conventional physical or rehabilitation therapy.

## Method

### Participants

Participant eligibility was based on the following inclusion and exclusion criteria. Inclusion criteria were the diagnosis of stroke, presence of post-stroke upper limb pain and spasticity after the acute phase, not being under physical or rehabilitation therapy before inclusion in the study, and provision of informed consent before participation (all participants gave written informed consent). Exclusion criteria were motor paresis, any metal implants, shunts or artifacts with a possible impact on current flow, diagnosis of other neurological or neuropsychiatric diseases, and any symptoms affecting understanding of the instructions and conduction of the study (aphasia, sensory deficit, etc.). The study was approved by the regional Ethics Committee for Biomedical Research (CEI), Huelva (PI 010/15), Spain, and conformed to the principles of the last version of the World Medical Association Declaration of Helsinki.

### Procedure

#### Transcranial Direct Current Stimulation

Anodal and sham tDCS were performed by a battery-driven constant-current stimulator (TCT Research Ltd. tDCS Stimulator, TST Kowloon, Hong Kong) (Wexler, 2015; Brennan et al., 2017) with conductive rubber electrodes placed between saline-soaked sponges. Each participant received 5 consecutive sessions of anodal and sham stimulation in randomized order, with a 3-week washout period between both stimulation conditions to avoid carryover effects. The anodal electrode was positioned over M1 of the affected hemisphere (the cathode over the homologous contralateral area), corresponding to the C3/C4 positions according to the international 10–20 EEG system for electrode placement (Klem et al., 1999; Herwig et al., 2003), and based on individual head measures. Stimulation in each session was applied for 20 min by two 5 × 4 cm (20 cm^2^) saline-soaked sponge electrodes at an intensity of 1.5 mA (0.075 mA/cm^2^). A gradual ramp up and down of stimulation for 10 s at the beginning and the end of stimulation, respectively, was programmed. The procedure was identical for sham condition, except that real current was only applied during the first and last 10 s to ensure the typical tDCS tingling sensation but avoid after-effects of stimulation. The electrodes were fixed onto the head by elastic rubber bands. After each intervention, participants were asked about any sensation related to tDCS application during stimulation. Participants were blinded to the respective stimulation conditions and were asked about a possible recognition of the specific condition in each case. A researcher not involved in data recording and analyses programmed each tDCS condition.

**FIGURE 1:**
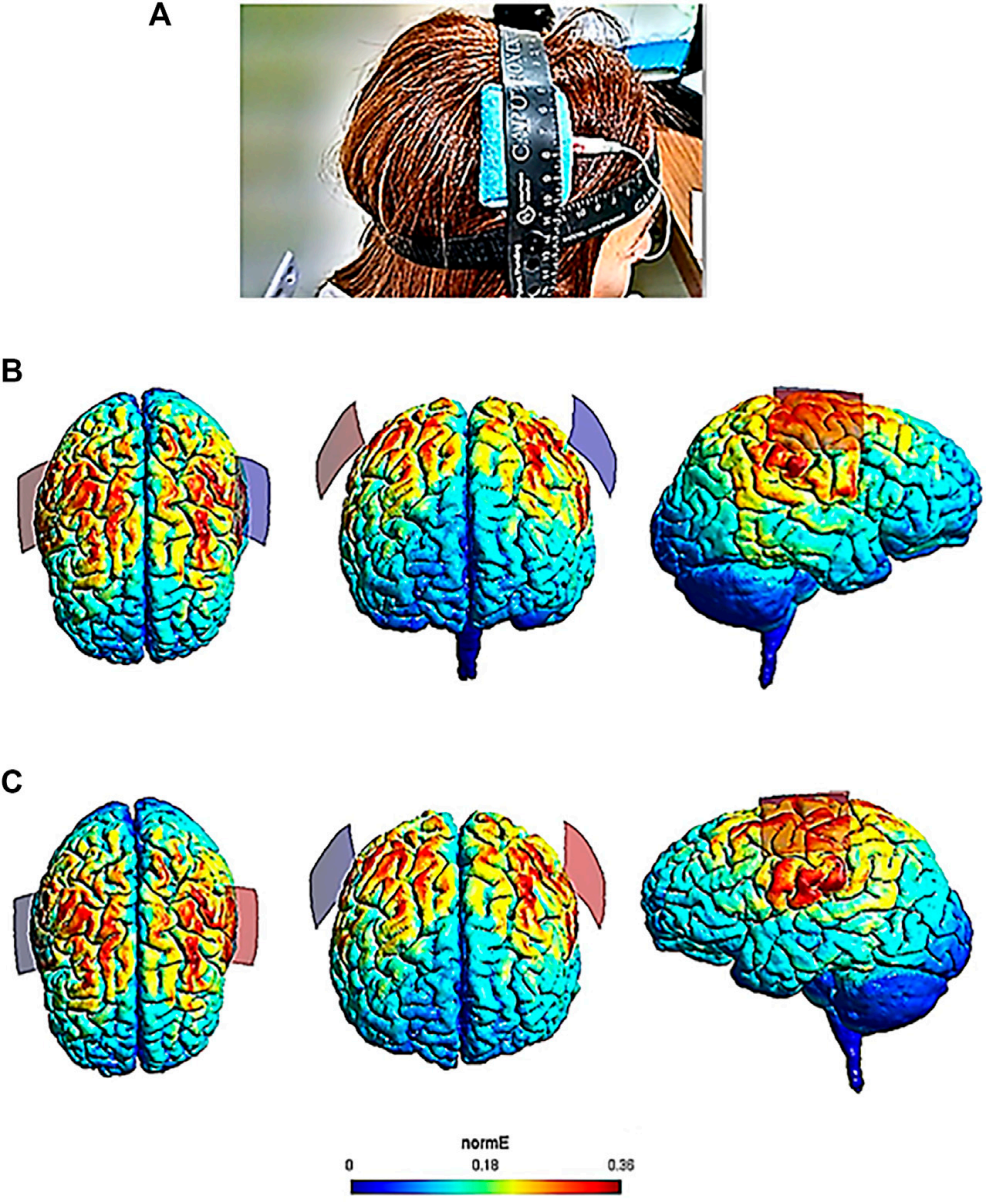
Cerebral electric field intensities calculated by the finite element method according to the tDCS electrode configuration used in this study. SimNIBS 3.1.2 software was used for modeling of the electric field. Panel **(A)** shows the electrode configuration used in one patient of the study (anode over the right M1 and cathode over the left M1, according to the international 10–20 EEG system). Panel **(B)** (anode over the right hemisphere) includes SimNIBS output brain images of the current flow from a dorsal, frontal and lateral view. Panel **(C)** (anode over the left hemisphere) includes SimNIBS output brain images from a dorsal, frontal and lateral view. The electric field (normE) intensity (V/m) is shown by the color bar. Brighter colors (higher numbers depicted in the color bar) indicate higher electric field intensity (0.36 V/m). Red and blue electrodes of the SimNIBS output brain images represent the anodal and cathodal electrode position, respectively.

SimNIBS 3.1.2 (Simulation of Non-Invasive Brain Stimulation) was used for modeling the intensity of the electric field induced by the different electrode configurations. SimNIBS is frequently used as computational modeling software to calculate the intensity of the electric field induced by tDCS (Saturnino et al., 2019). Figure 1 shows the electrode configuration used in this study, as well as the resulting electric field intensity calculated by the finite element method from this modeling tool.

#### Pain and Spasticity Measures

Pre-tDCS (before stimulation) and post-tDCS (after stimulation) pain measures were taken in each stimulation session for anodal and sham conditions. The Adaptive Visual Analog Scales of pain intensity and improvement (AVAS) (Marsh-Richard et al., 2009) were applied to obtain pain scores. AVAS provides separate values for self-perceived pain intensity and subjective pain improvement in successive measures. The Fugl-Meyer scale (Gladstone et al., 2002) was used to evaluate spasticity. The Fugl-Meyer scale is widely used to measure different dimensions of disability of stroke patients in the chronic phase. We used the joint mobility values of this scale to evaluate spasticity. These mobility measures were obtained for each patient at two time points, one before the first intervention (before anodal or sham stimulation), and the second one at the end of the fifth session (after anodal or sham stimulation).

### Statistical Analysis

Because of the limited number of patients who met the inclusion criteria of the study, and data distribution issues, ANOVAs or other parametric statistics to analyze between-subject data could not be conducted. Thus, the non-parametric Wilcoxon-Mann-Whitney Test, the most frequently used statistical analysis alternative to *t*-tests (Lumley et al., 2002; Dexter, 2013; Parker et al., 2020), was performed to compare the means between anodal and sham stimulation conditions of the AVAS values for pain intensity and improvement of each patient. In addition, pain intensity and pain improvement values were analyzed for each stimulation condition in each patient by the percentage of nonoverlapping data (PND) index. This is the most widely used index to assess the magnitude of therapeutic change in single case studies (Scruggs and Mastropieri, 2013). According to this index, values between 0 and 0.49 indicate that the intervention is not effective; values between 0.5 and 0.69 indicate uncertain effectiveness; values between 0.7 and 0.89 indicate clear effectiveness; and values between 0.9 and 1 indicate that the treatment is highly effective. Autocorrelations of anodal and sham overall data were calculated before by the autocorrelation function method (ACF) (Metcalfe and Cowpertwait, 2009) to rule out a possible serial dependency of data in each patient. Differences of percentage changes between pre- and post-anodal and sham stimulation conditions were calculated to analyze spasticity improvement values (increased mobility of the upper limb) of the Fugl-Meyer scale in each patient. This method has been previously described to analyze differences between percentages (Thunder et al., 1995). Also the average AVAS scores of pain improvement of the three patients were analyzed by percentage differences between pre-tDCS and post-tDCS in each stimulation condition (anodal vs. sham). The analyses were carried out by SPSS software (IBM SPSS Statistics V25.0).

## Results

### Patient Characteristics

Three stroke outpatients (2 women and 1 man; 43, 72 and 57 years old, respectively) recruited from the J.R. Jiménez Hospital (Huelva, Spain), with upper limb chronic post-stroke pain and spasticity but without motor paresis, met all inclusion criteria and voluntarily participated in this study. The interval between stroke onset and diagnosis and the start of the study was between 9 and 15 months.

### Patient Baseline Data

Considering the neuroimaging data, lesion size was similar in all patients, without structural M1 affection, but lesions were localized in different hemispheres (right hemispheric in two of the patients and left hemispheric in the third patient). Pain (AVAS mean values = 6.7) and spasticity (Fugl-Meyer mean values = 19) symptoms were moderate in all patients at the start of the study. The patients were not under rehabilitation therapy before participation or throughout the trial. Three or four weeks before the intervention, pain and spasticity symptoms of two patients were treated by botulinum toxin. Apart from this, no systematic pharmacological analgesic therapy was conducted in these patients at the start of the study. During the course of the study, the patients did not receive any other treatment.

### Outcome Data

According to the electric field modeling (Figure 1), the highest electric field intensities (0.36 V/m) corresponded to the cortical targets (left and right M1). The three patients reported tingling and itching sensations in both tDCS conditions, but no serious adverse effects were experienced. When they were asked, there was no certainty about the sessions in which they received real or sham stimulation.

The autocorrelation values of the ACF method for anodal and sham stimulation conditions indicated that there was no serial dependency of data for pain intensity and pain improvement in any of the patients (*p* > 0.05 in all cases). The Wilcoxon-Mann-Whitney test revealed significant differences between the pain intensity mean values of the anodal and sham tDCS conditions in all patients (*p* = 0.005, *p* = 0.007, *p* = 0.028, respectively), with reduced pain intensity values after anodal tDCS in patients 1 and 2. Table 1 shows the Wilcoxon-Mann-Whitney test results of pain intensity and pain improvement of the AVAS scores for the anodal and sham overall data of each patient. The PND index for pain intensity revealed that anodal tDCS was a highly effective intervention in patients 1 and 2 (PND = 1), and a clearly effective intervention in patient 3 (PND = 0.89). Values for sham tDCS in patients 1 and 3 were also PND = 1, but the median value for pain intensity of patient 1 in the sham condition was five times higher compared to the value of anodal tDCS, and the same median value for sham and anodal tDCS was found in patient 3 (Table 2). Table 2 depicts the results of the PND index for pain intensity and pain improvement AVAS values with respect to the anodal and sham overall data of each patient.

**TABLE 1:** Results of the Wilcoxon-Mann-Whitney Test conducted to compare the AVAS mean values of pain intensity and improvement obtained in the anodal and sham stimulation conditions in each patient.

AVAS	Mann-Whitney U	W of Wilcoxon	*Z*	*p*	Average range (anodal)	Average range (sham)
I P1	9.5	54.5	–2.828	0.005	6.06	12.94
PI P1	2	47	–3.526	<0.001	13.78	5.22
I P2	10.5	55.5	–2.687	0.007	6.17	12.83
PI P2	4.5	49.5	–3.288	0.001	13.5	5.5
I P3	22.5	67.5	–2.191	0.028	11.5	7.5
PI P3	21	66	–1.752	0.08	11.67	7.33

I, pain intensity; PI, pain improvement; P1-3, patient 1-3

**TABLE 2:** Results of the percentage of nonoverlapping data (PND) index for the pain intensity and pain improvement AVAS values regarding the anodal and sham overall data of each patient.

AVAS	PND index	BL value	Outcomes U/O BL	Mean	Median	Standard deviation	Minimum	Maximum
Pain intensity P1								
Anodal tDCS	1	8	9	1.44	1.0	1.59	0.0	5.0
Sham tDCS	1	8	9	4.22	5.0	1.72	2.0	6.0
Pain improvement P1								
Anodal tDCS	1	0	9	8.00	8.0	2.40	2.0	10.0
Sham tDCS	0.22	0	2	1.22	0.0	2.54	0.0	7.0
Pain intensity P2								
Anodal tDCS	1	10	9	4.44	4.0	2.01	1.0	7.0
Sham tDCS	0.33	7	3	7.44	7.0	1.59	5.0	9.0
Pain improvement P2								
Anodal tDCS	1	0	9	5.78	6.0	1.99	3.0	9.0
Sham tDCS	0.22	0	2	0.89	0.0	2.03	0.0	9.0
Pain intensity P3								
Anodal tDCS	0.89	2	8	0.56	0.0	0.73	0.0	2.0
Sham tDCS	1	2	9	0.0	0.0	0.00	0.0	0.0
Pain improvement P3								
Anodal tDCS	0.89	0	8	3.22	3.0	2.22	0.0	7.0
Sham tDCS	0.55	0	5	1.89	1.0	3.30	0.0	10.0

BL, baseline value; P1-3, patient 1–3; U/O, outcomes under (for pain intensity) or over (for pain improvement) the baseline values.

**FIGURE 2:**
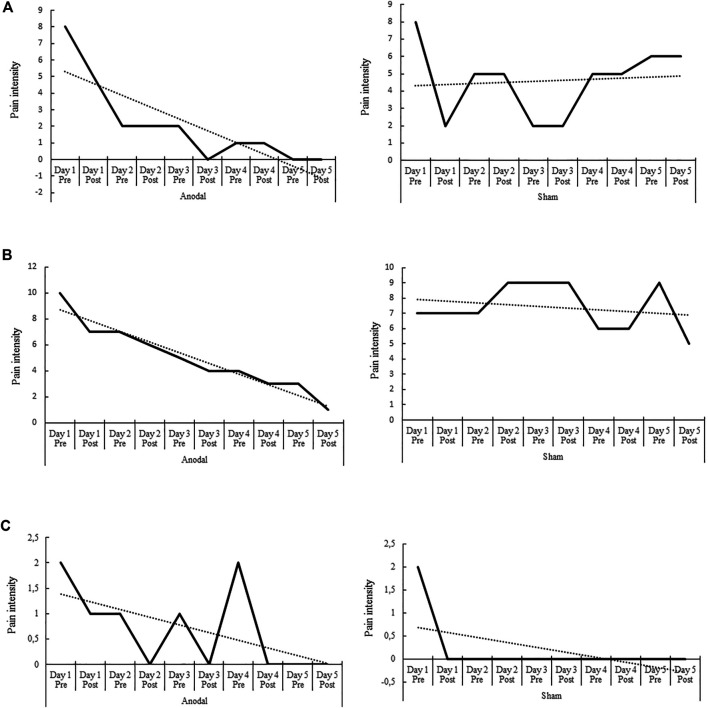
Pre- and post-anodal and sham tDCS values of pain intensity of the Adaptive Visual Analog Scales of pain intensity and improvement (AVAS) for each of the three patients **(A–C)** throughout the five intervention sessions (day 1–5). The dotted line indicates the data trend. Significant differences between pain intensity mean values of the anodal and sham tDCS conditions were found in each patient (*p* = 0.005, *p* = 0.007, *p* = 0.028, respectively), with reduced pain intensity values after anodal tDCS in patients 1 and 2.

**FIGURE 3:**
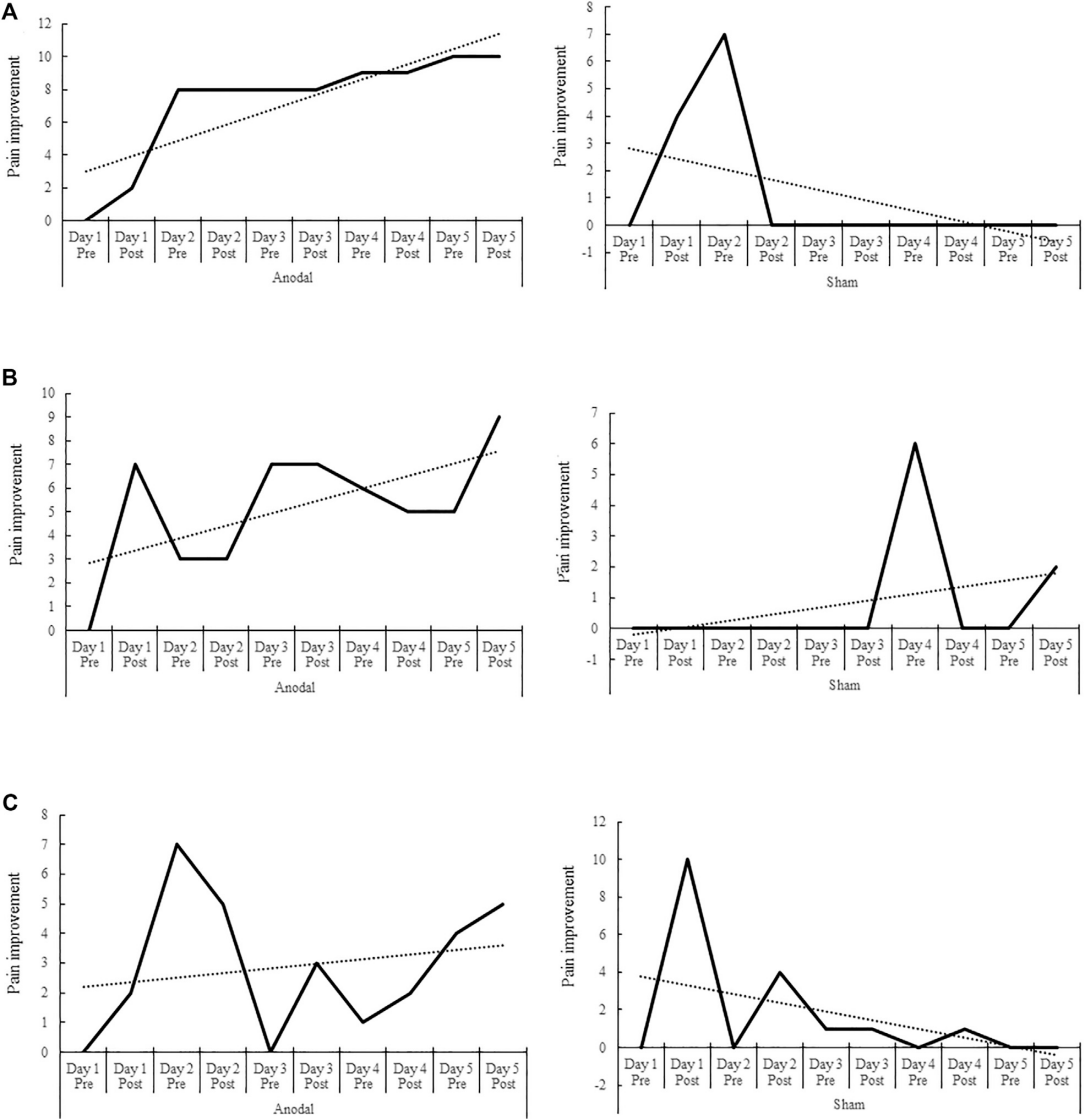
Pre- and post-anodal and sham tDCS values of pain improvement of the Adaptive Visual Analog Scales of pain intensity and improvement (AVAS) for each of the three patients **(A–C)** throughout the five intervention sessions (day 1–5). The dotted line represents the data trend. Significant differences between the pain improvement mean values of the anodal and sham stimulation conditions were found in patients 1 and 2, with superior improvements after anodal stimulation (*p* < 0.001 and *p* = 0.001, respectively). The differences between the mean values of the anodal and sham conditions in patient 3 were not significant, although there was a trend toward significance (*p* = 0.08).

The Wilcoxon-Mann-Whitney test revealed also significant differences between the pain improvement mean values of the anodal and sham tDCS conditions in patients 1 and 2, with superior improvements after anodal stimulation (*p* < 0.001, *p* = 0.001, respectively), but not in patient 3 (*p* = 0.08) (Table 1). The PND index for pain improvement revealed that anodal tDCS was a highly effective intervention in patients 1 and 2 (PND = 1), and a clearly effective intervention in patient 3 (PND = 0.89). The PND index for pain improvement after sham tDCS was smaller than 0.6 in all patients (PND = 0.22, 0.22, and 0.55, for patients 1, 2 and 3 respectively) (Table 2). Figures 2, 3 show the AVAS values of pain intensity and pain improvement, respectively, pre- and post-anodal and sham tDCS, for each patient throughout the five sessions.

**FIGURE 4:**
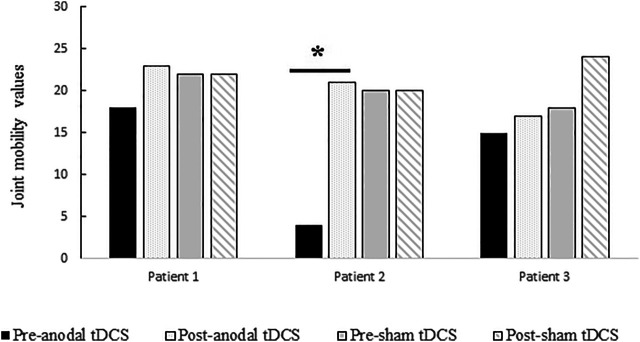
Fugl-Meyer joint mobility (spasticity) values of the three patients before the intervention (day 1, pre-anodal or sham tDCS) and after the fifth stimulation session (post-anodal or sham tDCS). Percentage differences were only significant between pre-anodal tDCS and post-anodal tDCS in patient 2 (*p* < 0.001).

Table 3 shows the percentage differences calculated for the pre- and post-anodal and sham tDCS conditions regarding the joint mobility (spasticity) values of the Fugl-Meyer scale in each patient. Figure 4 depicts the Fugl-Meyer joint mobility values of the three patients before the intervention (day 1, pre-anodal and sham tDCS) and after the fifth stimulation session (post-anodal and sham tDCS). In patient 1, the percentage of spasticity improvement after stimulation in relation to the highest score of the Fugl-Meyer scale was 11.37% in the anodal condition (*Z* = 1.06, *p* = 0.142) and 0% in the sham condition (*Z* = 0, *p* = 1). In patient 2, the percentage of spasticity improvement between pre- and post-stimulation in relation to the highest score of the Fugl-Meyer scale was 38.63% in the anodal condition (*Z* = 4.01, *p* < 0.001) and 0% in the sham condition (*Z* = 0, *p* = 1). In patient 3, the percentage of spasticity improvement between pre- and post-stimulation was 4.5% in the anodal condition (*Z* = 0.44, *p* = 0.328) and 13.63% in the sham condition (*Z* = 1.28, *p* = 0.100).

**TABLE 3:** Percentage differences calculated for the pre- and post-anodal and sham stimulation conditions with regard to the spasticity improvement values of the Fugl-Meyer scale in each patient.

	Anodal				Sham			
	pre	post	*Z*	*p*	pre	post	*Z*	*p*
Patient 1	18	23	1.06849	0.14265	22	22	–	–
Patient 2	4	21	4.01837	0.00003	20	20	–	–
Patient 3	15	17	0.4432	0.32881	18	24	1.28053	0.10018

## Discussion

In the present study we applied both anodal and sham tDCS over the affected M1 in multiple sessions (five consecutive days) in three patients who did not receive concurrent physical therapy. The overall results indicate a significant effect of the stimulation on pain in two patients after atDCS (Figures 2, 3), and a significant effect of atDCS on spasticity in one of the patients (Figure 4). The effect on pain intensity had a large size in two patients. In the third patient, a reduced pain intensity was also observed after anodal tDCS, but this effect was smaller, as compared to the other two cases, and was also observed in the sham condition. The known high inter-individual variability associated with the clinical, behavioral and cognitive effects of tDCS (Hsu et al., 2016; Katz et al., 2017; Falcone et al., 2018; Filmer et al., 2019) could explain these differences. The effects on pain intensity and pain improvement of atDCS were however relatively stable over time points in all patients, although the intervention had different effectiveness in each case, particularly in patient 3 (Figures 2, 3). Regarding the Fugl-Meyer scores of spasticity, the effect of atDCS was also heterogeneous since joint mobility after verum tDCS increased significantly only in patient 2, with nearly 40% improvement, as compared to the pre-intervention measures (38.63% of spasticity improvement, *p* < 0.001). A trend toward spasticity improvement was also observed after atDCS in patient 1 (11.37% of improvement, as compared to pre-tDCS), but this difference was not significant (*p* = 0.142) (Figure 4). These heterogeneous effects of the intervention may be caused by individual differences of the specific brain pathology and cortical re-mapping. The structural lesion of the patients was, although in different hemispheres, of similar size and location. However, post-stroke specific alterations of brain circuits and possible re-mapping were not evaluated in this study. The heterogeneity and variability of results could also be affected by baseline differences between both stimulation conditions. Nevertheless, the pre-tDCS pain intensity scores of the first day of intervention did not differ significantly between the anodal and sham conditions in any of the patients (Figure 2).

Other studies in which tDCS was applied over M1 have also found variability in results (Chew et al., 2015; Madhavan et al., 2016). To reduce inter-individual variability in the responses to stimulation, some adjustments of tDCS protocols have been suggested (Esmaeilpour et al., 2018; Khan et al., 2019). Personalized application of tDCS doses using computational models based on magnetic resonance imaging (Datta et al., 2012; Im et al., 2018; Esmaeilpour et al., 2020) and individual electric field modeling by finite element methods (Chew et al., 2015; Laakso et al., 2015; Evans et al., 2020) are current attempts to optimize the effect of stimulation and overcome inter-individual and intra-individual variability.

Overall, the results of the present study support the therapeutic potential of atDCS applied over M1 on chronic pain that has been reported in previous studies (Antal et al., 2010), and provide further evidence on specific effects of tDCS on post-stroke pain (Bae et al., 2014; Morishita and Inoue, 2016) and spasticity (Del Felice et al., 2016; Elsner et al., 2016). Post-stroke central pain involves an alteration of connections between the thalamus and M1. Considering that anodal stimulation increases M1 excitability (Nitsche and Paulus, 2000; Nitsche an Paulus, 2001; Nitsche et al., 2005), the effect of atDCS on pain in the present study could be attributed to an effective modulation of the activity of M1-thalamic connections. The increased excitability of M1 after atDCS was also likely effective to enhance activity of the corticospinal pyramidal system (Lang et al., 2004), thus reducing post-stroke spasticity due to downregulation of extrapyramidal activity. This effect was however less evident than the effect on pain, which suggests that the intervention had a differential influence on both neural mechanisms and their functions.

The analgesic effect of tDCS has also been explored in other chronic pain conditions such as phantom limb pain. A single session of anodal tDCS applied over the sensorimotor cortex region (S1/M1, with the target electrode positioned over C3 or C4), with concurrent imaginary movement of the missing hand, induced long-lasting phantom limb pain relief (Kikkert et al., 2019). In this protocol, the electrodes size was larger (5 × 7 cm), compared to our study (5 × 4 cm), and stimulation was applied simultaneously with a motor task. All of this might have resulted in a more efficient S1/M1 network stimulation and increased efficacy on chronic pain relief, which remains to be explored in more detail in future research.

Regarding safety and tolerability of the interventions, the patients of the present study did not report or experience any serious adverse effects during or after tDCS, and only the typical tingling and itching sensations over the scalp associated to this technique were reported. These effects were verbally mentioned by the three patients in both stimulation conditions, i.e., anodal and sham tDCS. This good tolerability is in accordance with the known excellent safety profile of tDCS (Brunoni et al., 2012; Bikson et al., 2016; Woods et al., 2016; Antal et al., 2017; Jackson et al., 2017a; Jackson et al., 2017b), which is also an important factor to consider this method for treatment in post-stroke patients (Stagg et al., 2012; Gomez Palacio Schjetnan et al., 2013; O’Shea et al., 2014; Fregni et al., 2015; Allman et al., 2016; Santos Ferreira et al., 2019).

The small number of participants is a limitation of this study. Upper and lower limb pain and spasticity are usually treated in post-stroke patients by physical rehabilitation (Veerbeek et al., 2014). We recruited three patients without any physical therapy in order to explore the genuine effect of the intervention, and this condition limited the number of available participants. Another limiting factor in studies investigating tDCS effects on post-stroke symptoms is concurrent pharmacological treatment (Alam et al., 2016a), which may add confounding factors to the results. The patients of the present study did not receive any systematic pharmacological therapy during the interventions. Two patients received botulinum toxin injection as pain treatment. However, the last injection was administered 3 or 4 weeks before intervention, thus reducing possible interferences of this treatment on tDCS effects. The variability regarding the pharmacological treatments of the patients included in tDCS studies may be a relevant factor in the explanation of heterogeneous results (Naro et al., 2017; Li and Morton, 2020). Therefore, control of the medication status in stroke patients will help to clarify the effects on post-stroke symptoms attributable to stimulation. On the other hand, the non-focality of tDCS has been considered a limiting factor of the anatomical specificity achieved by this stimulation method (Mikkonen et al., 2020), and for this reason a more focal stimulation over the target areas has been described in clinical studies using HD-tDCS (Richardson et al., 2015; Alam et al., 2016b). Nonetheless, lesions in stroke patients are not always well determined, and therefore focality might be difficult to stablish when treating these patients with tDCS. A last limitation of this study is that the pain measures were recorded before and after each of the five interventions, but a follow-up period was not implemented. To explore long-term effects of tDCS on limb pain and spasticity, and thus determine its clinical relevance, longer follow-up periods would be required in future studies.

## Conclusion

The treatment of motor symptoms in post-stroke patients has been investigated in previous studies by non-invasive brain stimulation methods, including tDCS (Elsner et al., 2017). Most tDCS studies in patients with stroke have applied stimulation to patients simultaneously with conventional rehabilitative treatments (Pollock et al., 2014), which makes difficult to establish the independent effect of this technique on motor recovery. This influence could also be relevant when evaluating tDCS effects on post-stroke pain and spasticity symptoms. In patients without concurrent physical therapy we found that five consecutive sessions of anodal tDCS over M1 of the affected hemisphere reduced pain and spasticity as evaluated by subjective (AVAS) and objective (Fugl-Meyer) scales. Therapeutic effects on pain were mainly found in two of the three patients included in this study. Spasticity was significantly improved only in one patient, and there was relevant inter-individual variability in the effects of stimulation. The implementation of medium and long follow-up periods in future studies with larger samples would provide relevant data on the after-effects of tDCS interventions in stroke patients, and thus help to determine the clinical usefulness of this approach.

## Data Availability

The raw data supporting the conclusion of this article will be made available by the authors, without undue reservation, to any qualified researcher.
